# IR76b-expressing neurons in *Drosophila melanogaster* are necessary for associative reward learning of an amino acid mixture

**DOI:** 10.1098/rsbl.2023.0519

**Published:** 2024-02-14

**Authors:** Naoko Toshima, Michael Schleyer

**Affiliations:** ^1^ Department Genetics of Learning and Memory, Leibniz-Institute for Neurobiology, Magdeburg 39118, Germany; ^2^ Institute for the Advancement of Higher Education, Hokkaido University, Sapporo 060-0810, Japan

**Keywords:** *Drosophila*, learning and memory, amino acids, ionotropic receptors, reward, punishment

## Abstract

Learning where to find nutrients while at the same time avoiding toxic food is essential for survival of any animal. Using *Drosophila melanogaster* larvae as a study case, we investigate the role of gustatory sensory neurons expressing IR76b for associative learning of amino acids, the building blocks of proteins. We found surprising complexity in the neuronal underpinnings of sensing amino acids, and a functional division of sensory neurons. We found that the IR76b receptor is dispensable for amino acid learning, whereas the neurons expressing IR76b are specifically required for the rewarding but not the punishing effect of amino acids. This unexpected dissociation in neuronal processing of amino acids for different behavioural functions provides a study case for functional divisions of labour in gustatory systems.

## 1. Introduction

All animals must find essential nutrients and avoid toxic food. Learning about cues that predict the presence of food sources or toxins is therefore a critical evolutionary advantage. Here, we report on the roles of a particular set of gustatory sensory neurons in *Drosophila melanogaster* larvae for associative learning of amino acids, the building blocks of proteins and thus one of the most important nutrients.

Previous studies found that several individual amino acids are innately attractive for larvae and can act as rewards in associative learning [1–3]. A mixture of all 20 canonical amino acids, surprisingly, turned out to act simultaneously as a reward and a punishment [4]. It was shown before that reward memories in larvae are only behaviourally expressed in the absence of the trained reward, and punishment memories are only in the presence of the trained punishment [1,5–8]. In the case of the amino acid mixture, reward memory was observed when the animals were tested in the absence, and punishment memory when the animals were tested in the presence of the amino acid mixture [4]. Such a ‘push–pull’ behavioural organization may help the animals to find an optimal concentration of amino acids.

Little is known about the neuronal circuits underlying amino acid sensing and processing [9]. In adults, both sweet-sensing and bitter-sensing gustatory receptor neurons have been shown to respond to amino acids, dependent on different combinations of gustatory and ionotropic receptors [10]. Specifically, the ionotropic receptor [11] IR76b is broadly expressed in external and pharyngeal taste neurons in *D. melanogaster* (larvae [12–14], adults [15,16]), and is required for innate preference or avoidance of amino acids (larvae [2], adults [10,16]) as well as other appetitive and aversive tastants [17–19]. The IR76b+ neurons physiologically respond to several single amino acids (larvae [2], adults [10,16]) and yeast [20].

However, it remains open whether IR76b, or the neurons expressing it, are also involved in learning about amino acids. Previous studies demonstrated that this is not a trivial question: in the case of the bitter tastant quinine, for example, larval GR66a+ neurons were found to be required specifically for innate avoidance but not for learning [21]. In adults, PPK28 expression was necessary for learning but not for innate preference of water [22].

Here, we ask whether the gustatory pathways to regulate the rewarding and punishing effects of amino acids are shared or separated. Specifically, we investigate the necessity of IR76b+ neurons for forming both reward and punishment memories with respect to amino acids, and the potential of artificial activation of IR76b+ neurons for inducing memories.

## 2. Material and methods

### (a) Animals

*Drosophila melanogaster* were maintained on standard medium at 25°C and under a 12 h : 12 h light : dark cycle except the larvae for the optogenetic experiments, which were raised in darkness throughout. Third-instar larvae (5 days after egg laying) were collected from the medium, briefly rinsed with tap water and used for behavioural tests. We used *IR76b-Gal4* (Bloomington: 41730), a strain that has been shown to express in the gustatory, but not the olfactory sensory organs in larvae [14]. This driver strain was crossed to *UAS-ChR2-XXL* (Bloomington: 58374) for the activation experiments, and to either *UAS-Kir2.1::GFP* [23] or *UAS-GtACR1* [24] for the silencing experiments. Driver controls were obtained by crossing *IR76b-Gal4* to *w*[1118] as the control for *UAS-ChR2-XXL* and *UAS-GtACR1*, or *Canton S* as the control for *UAS-Kir2.1::GFP*. Effector controls were obtained by crossing UAS strains to *w*[1118]. We further used the mutant strain *IR76b*[1] (Bloomington: 51309) and the rescue strain *IR76b-Gal4, UAS-IR76b; IR76b*[1] as described in [2]. To obtain heterozygous mutant flies, *IR76b*[1] was crossed to *w*[1118].

### (b) Chemicals

We diluted *n*-amyl acetate (AM) diluted 1 in 20 in paraffin oil and filled into custom-made Teflon containers (10 µl). The preparation for a 20 amino acid mixture followed the previously reported procedure [4]. Briefly, 20 amino acids were mixed into 1% agarose at concentrations of 0.5 mM for each amino acid (10 mM in total). For a list of all chemicals, see table 1.

**Table 1.** List of all chemicals used in this study.

chemical	CAS number	supplier
*n*-amyl acetate	628-63-7	Merck (Darmstadt, Germany)
paraffin oil	8042-47-5	AppliChem (Darmstadt, Germany)
agarose	9012-36-6	Roth (Karlsruhe, Germany)
l-alanin	56-41-7	Sigma-Aldrich (Seelze, Germany)
l-arginine	74-79-3	Sigma-Aldrich
l-asparagine	70-47-3	Sigma-Aldrich
l-aspartic acid	56-84-8	Sigma-Aldrich
l-cysteine	52-90-4	Sigma-Aldrich
l-histidine	71-00-1	Sigma-Aldrich
l-isoleucine	73-32-5	Sigma-Aldrich
l-leucine	61-90-5	Sigma-Aldrich
l-lysine	56-87-1	Sigma-Aldrich
l-glutamine	56-85-9	Sigma-Aldrich
l-glutamic acid	56-86-0	Sigma-Aldrich
l-glycine	56-40-6	Sigma-Aldrich
l-methionine	63-68-3	Sigma-Aldrich
l-phenylalanine	63-91-2	Sigma-Aldrich
l-proline	147-85-3	Sigma-Aldrich
l-serine	56-45-1	Sigma-Aldrich
l-threonine	72-19-5	Sigma-Aldrich
l-tryptophan	73-22-3	Sigma-Aldrich
l-tyrosine	60-18-4	Sigma-Aldrich
l-valine	72-18-4	Sigma-Aldrich

### (c) Associative learning

The experiments followed a standard paradigm [25,26], slightly modulated to use only one instead of two odours. This one-odour paradigm is well established and usually yields similar levels of learning as the two-odour version [4,6–8,23,27]. Petri dishes of 90 mm diameter were covered with either pure 1% agarose or 1% agarose with amino acids mixture.

For the experiments with the effector *UAS-Kir2.1*, groups of approximately 30 transgenic larvae were trained to associate the amino acid mixture (+) and AM odour. Animals were allowed to freely move on an amino acid-filled Petri dish equipped with two odour containers filled with AM for 2.5 min. Afterwards, animals were transferred to a pure Petri dish, equipped with two empty odour containers (EM), and left for another 2.5 min (paired training; AM+/EM). A second group of larvae were trained reciprocally, i.e. they experienced the amino acid mixture and AM during separate trials (unpaired training; AM/EM+) (the sequence of training trials was reversed in half of the cases: EM/AM+ and EM+/AM, respectively). After repeating such training for three cycles, animals were transferred to either a pure or amino acid mixture-filled Petri dish equipped with an AM container on one side and an EM container on the other side. After 3 min the number of larvae on the AM side (#AM), on the EM side (#EM) and in a 10 mm wide middle zone was counted and the preference for AM was calculated:

$$\mathrm{AM}\;\mathrm{Pref}=\frac{\#\mathrm{AM}-\#\mathrm{EM}}{\#\mathrm{Total}}.$$

Positive AM Pref scores indicate attraction, negative scores aversion to the odour.

To quantify associative memory, the performance index (PI) was calculated from the AM Pref scores of the reciprocally trained groups:

$$\mathrm{PI}=\frac{\mathrm{AM}\;\mathrm{Pref}(\mathrm{AM}+/\mathrm{EM})-\mathrm{AM}\;\mathrm{Pref}(\mathrm{EM}+/\mathrm{AM})}{2}.$$

Thus, positive and negative PI scores indicate appetitive and aversive associative memory, respectively.

Optogenetic silencing experiments with *UAS-GtACR1* as effector were performed in an analogous way, using an optogenetic set-up as previously reported [23], using a custom-made LED light table (520 nm LED, 2000 µW cm^−2^; Solarox), with a frosted Plexiglas cover for diffusion. Green light was presented throughout the training and the test, otherwise training and testing procedures were the same as above.

For optogenetic activation experiments with *UAS-ChR2-XXL* as effector, larvae received odour–light associative training in the same set-up but with blue light (470 nm LED, 120 µW cm^−2^; Solarox) in groups of about 20 animals. Otherwise training and testing procedures were the same as described; namely, paired training groups received AM odour together with blue light stimulation, while unpaired training groups received the odour and the light stimulation separately. The test was performed either in darkness or under blue light, and in the absence or the presence of the amino acid mixture in darkness.

### (d) Innate odour preference

Innate odour preference was measured by collecting animals and then directly testing their preference for the odour as described above, using pure test Petri dishes. AM Pref scores were calculated according to the above-mentioned equation. Optogenetic transgenic larvae were tested in the previously described set-up under darkness or light. Non-optogenetic experiments were conducted under room light.

### (e) Statistics

Two-tailed non-parametric tests were applied throughout, using Statistica 13.0 (StatSoft software, Hamburg, Germany). For multiple-group comparisons, Kruskal–Wallis tests were applied, and if significant, were followed by pairwise comparisons with Mann–Whitney *U*-tests (MWU). One-sample sign tests (OSS) were applied to test significant differences from zero. When multiple tests were applied in one experiment, Bonferroni–Holm corrections were used to maintain error rates below 5%.

## 3. Results

### (a) IR76b+ neurons are partially responsible for the rewarding, but not for the punishing effect of the amino acid mixture

**Figure 1.**
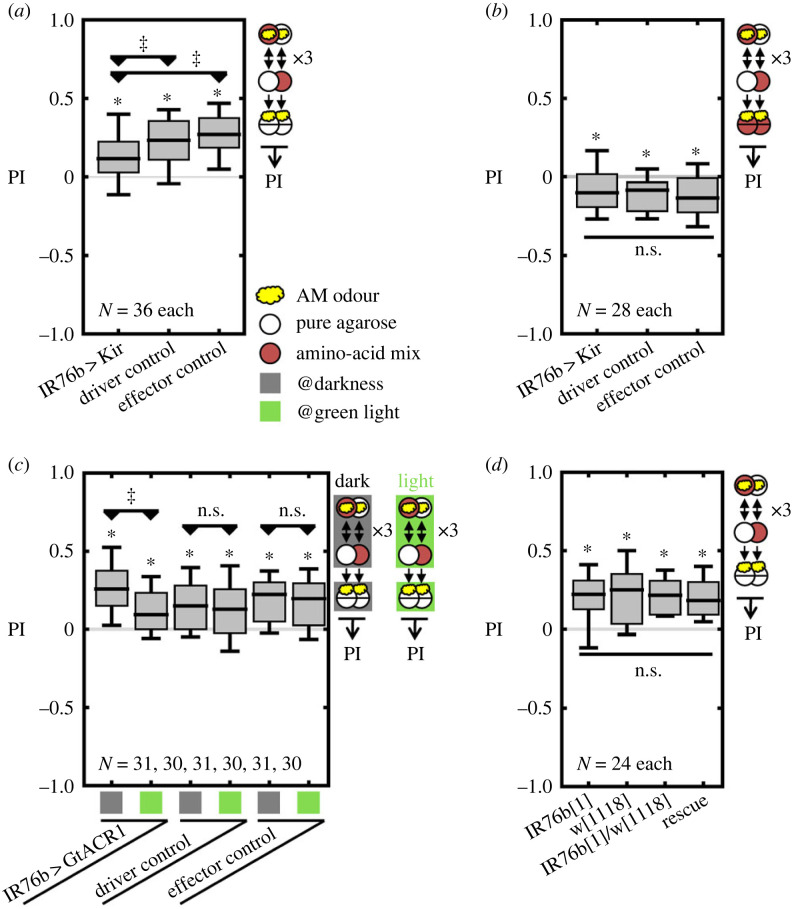
IR76b+ neurons are necessary for signalling the rewarding but not the punishing effect of an amino acid mixture. (*a*) After odour–amino acid mixture learning experiments, reward memory was significantly reduced in the animals with silenced IR76b+ neurons relative to two genetic controls. (*b*) Punishment memory was intact in the experimental group relative to two genetic controls. (*c*) To acutely silence IR76b+ neurons, optogenetic experiments were performed either in darkness or green light. Reward memory was significantly reduced under green light only in animals expressing GtACR1 in IR76b+ neurons. (*d*) Experiments were performed using the *IR76b*[1] mutant. Reward memory was intact relative to a *w*[1118], a heterozygous and a rescue control. In all cases, the innate AM preferences were mostly unaffected by the experimental manipulations (electronic supplementary material, figure S4). Data are presented with the median as middle line, the quartiles as box boundaries and the 10/90% quantiles as whiskers. Significant difference from zero (OSS tests): *, significant pairwise difference (MWU): ^‡^, Underlying AM preferences are shown in electronic supplementary material, figure S1. All raw data and exact values of statistical tests are given in electronic supplementary material, table S1.

First, we asked whether IR76b+ neurons are involved in associative amino acid learning. Larvae were trained to associate an odour and the amino acid mixture. When tested in the absence of the mixture, genetic controls showed a reward memory, which was partially but significantly reduced when the IR76b+ neurons were silenced (figure 1*a*; electronic supplementary material, figure S1*a*). When larvae were tested in the presence of the mixture, punishment memory was intact in the experimental group compared to the genetic controls (figure 1*b*; electronic supplementary material, figure S1*b*). The phenotype in reward memory was confirmed by acute optogenetic silencing of IR76b+ neurons with *UAS-GtACR1* (figure 1*c*; electronic supplementary material, figure S1*c*). These results imply that IR76b+ neurons are required only for the rewarding but not the punishing effect of the amino acid mixture.

Next, we asked whether the expression of the *IR76b* receptor gene is necessary for the associative amino acid mixture reward learning. Towards this end, we trained the *IR76b*[1] mutant larvae to associate the amino acid mixture and the odour. Compared to the genetic controls, *IR76b*[1] larvae showed an intact reward memory (figure 1*d*; electronic supplementary material, figure S1*d*), suggesting that although IR76b+ neurons are necessary for associative amino acid reward learning, IR76b expression in the IR76b+ neurons is not.

### (b) Both reward and punishment memories can be established through IR76b+ neurons

**Figure 2.**
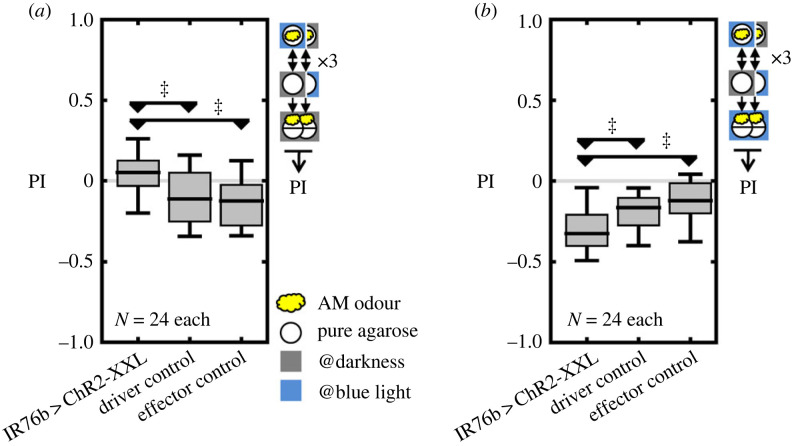
Optogenetic activation of IR76b+ neurons can be both rewarding and punishing. (*a*) After training with odour and IR76b+ activation, reward memory was observed compared to the genetic controls when tested in darkness, (*b*) whereas punishment memory was observed when IR76b+ neurons were activated during the test. In all cases, the innate AM preferences were mostly unaffected by the experimental manipulations (electronic supplementary material, figure S4). Further details as in figure 1. OSS tests were not applied since genetic controls show slightly negative memories, potentially because blue light also works as punishment. Underlying AM preferences are shown in electronic supplementary material, figure S2.

Given the importance of IR76b+ neurons for associative amino acid reward learning, we wondered whether artificially induced activity of IR76b+ neurons would act as a reward itself. Groups of larvae expressing the optogenetic effector *ChR2-XXL* in IR76b+ neurons were trained to associate the odour with blue light presentation, and thus activation of IR76b+ neurons. Relative to the genetic controls, these larvae exhibited significantly more positive memory scores, indicating that optogenetic activation of IR76b+ neurons indeed was rewarding (figure 2*a*; electronic supplementary material, figure S2*a*). As discussed above, reward memories are usually only revealed in the absence of the reward. Therefore, we repeated the experiment, but tested the animals in the presence of blue light and thus with activated IR76b+ neurons. In a previous study such optogenetic activation of rewarding neurons mimicked the presence of a reward and abolished the expression of the reward memory [8]. Strikingly, under these conditions we observed significantly negative memory scores compared to the genetic controls (figure 2*b*; electronic supplementary material, figure S2*b*), a hallmark of aversive odour—taste memories which are preferentially expressed in the presence of the respective taste punishment [1,5–8]. Finally, with the amino acid mixture present in the test, we found no significant memory expression. From these results, we cannot discern whether the amino acid mixture abolished the reward memory expression, or allowed a punishment memory expression that cancelled out the reward memory (electronic supplementary material, figure S3). In either case, the results suggest that the memories induced by activation of IR76b+ neurons were partially about amino acids.

In all experiments, the innate AM preferences were mostly unaffected by the experimental manipulations (electronic supplementary material, figure S4).

Taken together, these results indicate that optogenetic activation of IR76b+ neurons can induce both associative reward and punishment memories, being revealed dependent on the testing conditions.

## 4. Discussion

**Figure 3.**
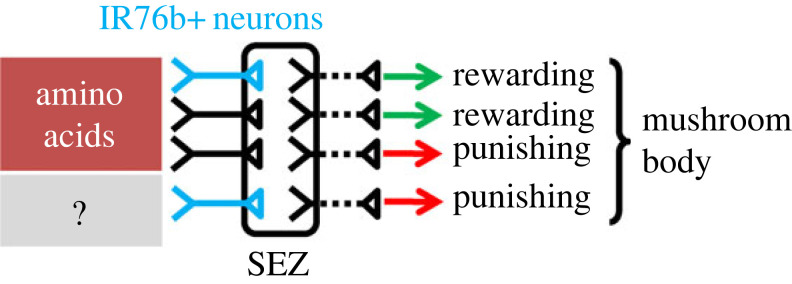
Schematic model. Amino acids have a rewarding and a punishing effect. The rewarding effect is partially signalled by IR76b+ neurons (blue), partially by unidentified IR76b+ neurons (black) (figure 1*a,c*). IR76b+ neurons are sufficient for a punishing effect (figure 2*b*), but not required for the punishing effect of amino acids (figure 1*b*). It remains open whether the punishing effect of IR76b+ neurons concerns amino acids or unknown stimuli (?). All taste sensory neurons carry their signals to the suboesophageal zone (SEZ), and potentially via several synapses to the mushroom body, the insects' memory centre.

In this study, we investigated the roles of IR76b+ neurons for the associative amino acid learning, and revealed several cases of unexpected complexity (figure 3):

1. We found that the IR76b receptor was dispensable for amino acid reward learning, but the activity of the IR76b+ neurons was not (figure 1). These results were unexpected, since IR76b was suggested to work as a general co-receptor for other IRs in the adult olfactory system [28]. Notably, IR76b+ neurons express a variety of other receptor genes in adults [13,15]. Therefore, our result suggests that other receptors within the IR76b+ neurons are responsible for the rewarding effect of amino acids without requiring IR76b as a co-receptor.
2. In addition, IR76b+ neurons turned out to be required for signalling only the rewarding, but not the punishing effect of the amino acid mixture (figure 1*a−c*). This does not exclude the possibility that IR76b+ neurons are involved also in signalling the punishing effect to some extent, but we need to assume another, IR76b-negative sensory pathway. These results support the notion that an amino acid mixture induces parallel memories of opposite valence that can be independently expressed, rather than a single memory that is valued positive or negative depending on the current situation (for a detailed discussion, see [4]). Our results suggest that the IR76b+ neurons are part of the neuronal pathway for forming the amino acid reward memory. Similar parallel memories of opposite valence were previously reported also in adults [29,30]. Notably, some reward memory remained upon silencing the IR76b+ neurons (figure 1*a,c*). The remaining memory may be based either on an IR76b-independent taste pathway, or on post-ingestive nutrition-sensing of the amino acids [31].
3. Although IR76b+ neurons are required only for the rewarding effect of amino acid mixtures, the activation of these neurons induces both rewarding and punishing effects (figure 2*a,b*). There are two potential explanations for this result: either both the rewarding and punishing effects induced by activating IR76b+ neurons indeed relate to amino acids, and the IR76b+ neurons are sufficient and required for the rewarding effect, but not required for the punishing effect; or the IR76b+ neurons mediate a punishing sensation of some unknown stimulus. Notably, in adult flies, IR76b is involved in sensing of aversive high sodium taste [19] and is co-expressed with GR66a in some presumably bitter-sensing neurons [10,15]. Whether this is the case also in larvae is not known yet. In any case, to the best of our knowledge this result is the first case of showing that artificial activation of sensory neurons can induce parallel appetitive and aversive memories in *Drosophila*.

Taken together, our study provides an example of how many aspects of the gustatory system are still not well understood and much more complex than anticipated (see also [32]), and uncovers unexpected complexity in how the larval gustatory periphery is wired up with the brain interneurons that confer valence. This, in a model organism with few neurons and excellent genetic tools, is an excellent study case to investigate the details of such division of labour in future research.

## Data Availability

All raw data are included in electronic supplementary material, table S1 (electronic supplementary material, file S6). The data are provided in the electronic supplementary material [33].
